# Protein modeling and molecular dynamic studies of two new surfactant proteins

**DOI:** 10.1186/1758-2946-5-S1-O2

**Published:** 2013-03-22

**Authors:** Felix Rausch, Wolfgang Brandt, Martin Schicht, Lars Bräuer, Friedrich Paulsen

**Affiliations:** 1 Department of Bioorganic Chemistry, Leibniz Institute of Plant Biochemistry, Weinberg 3, 06120 Halle, Germany; 2 Department of Anatomy II, University of Erlangen-Nuremberg, Universitätsstr. 19, 91054 Erlangen, Germany

## 

Surfactant proteins are of major importance for the stability and flexibility of lipid systems like the lung surfactant or the tear film. They can support the adsorption of phospholipids into a layer or specifically influence the surface tension of a lipid surface [1]. To fulfil these functions, they are described to be highly posttranslationally modified. Furthermore, immunological functions were described for some of the already known surfactant proteins SP-A, SP-B, SP-C and SP-D [2]. For that reason, they are of great interest in the investigation of diseases like the "acute respiratory distress syndrome" (ARDS) or the "dry eye syndrome" (DES). Recently sequences of two putative surfactant proteins, called SP-G and SP-H, were identified. To get insights into the function of SP-G and SP-H, protein structure models were generated, including predictions for posttranslational modifications. These models successfully guided the design of antibodies for the localization of SP-G and SP-H in different human tissues, including lung and ocular system. Both protein models were transferred into a lung surfactant model system consisting of a dipalmitoyl¬phosphatidyl¬choline monolayer. MD simulations over 50 ns were performed with GROMACS starting from different orientations of the protein models. During these simulations, it was possible to track the accumulation of the proteins to the lipid layer and observe the interactions between protein surface and lipid head groups on an atomic scale. The results show that SP-G and SP-H indeed are capable of surface-regulatory properties. Furthermore, it is evident that the interaction between proteins and lipid systems is highly dependent on the present posttranslational modifications. In combination with further experimental work, these simulations can help to determine the functions of SP-G and SP-H *in vivo *and may support the development of new therapies for ARDS and DES.
